# Not a Quiet Place: Understanding Noise Level in a Newborn Intensive Care Unit (NICU) and Its Relation with Newborn’s Vital Parameters, a Pilot Feasibility Study

**DOI:** 10.3390/children12060757

**Published:** 2025-06-11

**Authors:** Silvia Rossi, Alessia Salvatore, Giulia Ottonello, Ilaria Artuso, Roberta Da Rin Della Mora, Simona Serveli, Silvia Scelsi

**Affiliations:** Direction of Health Professional, IRCCS Istituto Giannina Gaslini, 16147 Genova, Italy; silviarossi@gaslini.org (S.R.); alessiasalvatore@gaslini.org (A.S.); ilariaartuso@gaslini.org (I.A.); robertadarindellamora@gaslini.org (R.D.R.D.M.); simonaserveli@gaslini.org (S.S.); silviascelsi@gaslini.org (S.S.)

**Keywords:** newborn intensive care units (NICU), noise, vital signs, infant, premature, tachycardia, nurses, pediatric, nursing care

## Abstract

**Background/Objectives:** Adaptation to extrauterine life is challenging for preterm newborns. Environmental stimuli, such as noise, can lead to adverse health outcomes, causing instability of vital parameters and impairment of neurodevelopment. The American Academy of Pediatrics recommends a maximum environmental noise level of 45 decibels (dB) or less in the NICU. The study’s primary aim was to describe environmental noise in a neonatal intensive care unit and to analyze potential associations between noise and vital parameters of preterm newborns, including heart rate, respiratory rate, and oxygen saturation levels. **Methods**: A pilot observational feasibility study was conducted in a level III NICU. Sound levels and vital parameters were recorded over four hours for each preterm newborn. Confounding variables were controlled. Data were analyzed using descriptive statistics, Kendall’s τ-b, and logistic regression analysis. Ethical approval and parental consent were obtained. **Results**: The average environmental noise level was consistently above 45 dB. Six patients were enrolled, and 22 recordings (ranging in length from 1 to 4 h) were performed. Data adjusted for confounding variables show a statistically significant Kendall’s correlation between heart rate and decibels (τ-b = 0.89, *p* = 0.003, n = 520), suggesting a monotonous crescent tendency between these two variables, although the relationship is not strong. The logistic regression model indicates that the odds ratio (OR) for decibels related to tachycardia is 1.066, meaning that for each 1 dB increase, the probability of tachycardia rises by 6.6% (*p* < 0.001). Conversely, the OR for respiratory rate is 0.959, suggesting that for each unit increase in respiratory rate, the probability of tachycardia decreases by approximately 4.1% (*p* < 0.001). **Conclusions**: The study reveals that the mean environmental noise level in the NICU consistently exceeds the recommended safety level. Decibels are one of the significant variables contributing to the likelihood of tachycardia, and an increase in decibels has a significant effect on this, but it is not the only one. Further analysis of a larger sample is needed.

## 1. Introduction

In 1994, the American Academy of Pediatrics (AAP) published an article highlighting the potentially dangerous effects of excessive environmental noise on the development and well-being of newborn infants. Since then, numerous studies have reported on the real effects of noise on newborns, especially preterm newborns, and on the various improvement projects aimed at reducing environmental noise in Newborn Intensive Care Units (NICUs) to below 45 decibels (dB), as recommended by the AAP [1].

The improvement projects have attempted to reduce mean environmental noise levels in various NICUs around the world, ranging from the use of earplugs to the implementation of rest periods to strategies such as staff education, environmental modifications (i.e., the introduction of single-family rooms), and the use of sound-absorbing materials [2,3,4,5,6,7,8]. Despite these studies, environmental noise remains a significant issue today, particularly when no architectural changes have been made to NICU rooms [9].

Common sources of environmental noise in NICUs include alarms and beeps from medical equipment such as ventilators, monitors, and infusion pumps, which can range from low hums to loud alerts. Additionally, the devices themselves—such as high-flow nasal cannula (HFNC) systems, continuous positive airway pressure (CPAP) machines, and fans integrated into incubators—generate constant background noise that significantly contributes to the overall sound environment. Conversations between healthcare professionals, although essential for patient care, further increase the acoustic load. Addressing all of these sources of noise is essential to promoting a quieter and more developmentally supportive NICU environment.

Research has shown that increased environmental noise is correlated with increased sympathetic nervous system activity, resulting in significant increases in heart rate, respiratory rate, and blood pressure, as well as a decrease in oxygen saturation [10,11,12,13]. Some studies have attempted to explain these relationships in detail, demonstrating an increase in neonatal heart rate when noise levels exceed 56 dB [14] or a significant increase in heart rate accelerations at 100 dB [15]. However, a lack of consensus remains on the precise effects of noise on neonatal vital parameters, particularly due to the extreme clinical heterogeneity of this population.

The study presented here is a pilot observational feasibility study conducted in the same setting as a larger Randomized Controlled Trial (RCT) [16] that addresses this critical issue in NICUs.

This pilot study aimed to investigate the relationship between environmental noise levels (measured in decibels) and fluctuations in neonatal vital parameters, specifically heart rate, respiratory rate, and oxygen saturation. To achieve this, we implemented a novel data collection approach that combines synchronized video recordings of vital signs with punctual measurements of environmental noise—an approach not previously applied in this specific context.

## 2. Materials and Methods

We conducted a pilot observational feasibility study on preterm newborns admitted to a level III NICU in a large pediatric hospital in Italy in July 2023 to understand the relationship between fluctuations in vital parameters (heart rate, respiratory rate, and oxygen saturation) and environmental noise.

In the published protocol [16], it was indicated that preterm newborns were included if they were ≥31 weeks of gestation and without major congenital or cerebral anomalies (e.g., intraventricular hemorrhage, brain insult, meningitis, airway abnormalities, etc.).

Eligible preterm newborns were enrolled using a convenience sampling approach after parental or guardian information and consent were obtained. All preterm newborns admitted to the NICU during the study period who met the inclusion criteria were considered for participation in the study. However, due to logistical constraints—including limited equipment (only two video cameras and two calibrated sound level meters were available) and a small, dedicated research team—it was only possible to enroll a maximum of two infants at a time. Enrollment was therefore performed sequentially based on the simultaneous availability of eligible patients and study resources.

The first period of this study was dedicated to registered environmental noise, excluding preterm newborn enrollment, to establish a baseline of environmental noise that would enable us to understand how our units perform in relation to the AAP recommendation. The second period was dedicated to the enrolment of preterm newborns and data collection.

### 2.1. Setting

The study was conducted in a 22-bed level III neonatal intensive care unit (NICU), organized into four open-bay rooms. No formal protocols or guidelines for noise control are currently in place in this unit. The first room, designated as the High-Care Room, includes six patient units, one of which is reserved explicitly for extremely preterm infants or those with severe infections. The second, the Medium-Care Room, contains eight patient units. The third and fourth rooms are designated as Low-Care Rooms, each accommodating four patient units.

### 2.2. Procedures

#### 2.2.1. Environmental Measurements

Environmental noise was recorded using two professional sound level meters (PCE-322A), each calibrated prior to use according to the manufacturer’s instructions using the standard calibration procedure recommended for the device.

To establish baseline environmental noise levels across the NICU, sound level meters were placed at different stations in each of the four rooms. Recordings were conducted during all shifts (morning, afternoon, and night), both inside the incubators and at the bedside. Each session lasted two hours.

Then, during the phase of patient enrollment, appropriately calibrated sound level meters were used to record environmental noise, either inside the incubator or at the bedside, depending on the preterm newborn’s accommodation. For preterm newborns in closed incubators, the sound level meter was placed inside the incubator, close to the newborn’s ear. For those in open cribs, the sound level meter was positioned approximately 30 cm from the newborn’s head on the bedside table of the patient unit. Each recording lasted 4 h.

#### 2.2.2. Vital Parameters Data Collection

Heart rate, oxygen saturation, and respiratory rate were obtained from the multiparameter monitor used in routine clinical practice, as specified in the study protocol [16]. These values, displayed on the monitor screen, are calculated in real-time by proprietary algorithms. As we did not have access to the raw physiological signals, the recorded values reflect processed outputs that may include time lags or smoothing effects inherent to the monitor’s software. This aspect represents a methodological limitation, particularly in time-sensitive correlations with environmental stimuli.

Since it was not possible to download data directly from the monitor, two video cameras were placed either outside the incubator or near the bed to record the monitor displaying the infant’s vital signs. To ensure privacy, only the monitor screen was recorded, without capturing any other part of the patient or the environment. The cameras were synchronized by date and time to allow comparison with environmental noise data collected via sound level meters. The researcher then analyzed the video recordings to extract and enter the data. Prior to video analysis, several meetings were held within the research group to ensure the reliability of data extraction and database entry. The methods were discussed, and some methodological doubts were resolved before the start of this phase.

All data (demographics, environmental noise, vital parameters, and confounding variables) were entered into a database created using Microsoft Excel software. We recorded the values of dB, heart rate, respiratory rate, and oxygen saturation at fixed 15 min intervals. Additionally, we recorded every time a clinically relevant event occurred—specifically, episodes of tachycardia (heart rate > 180 bpm), tachypnea (respiratory rate > 60 breaths per minute), or desaturation (oxygen saturation < 90%). No bradycardia events were observed; therefore, none were included in the analysis. The 15 min time points were chosen to provide a regular overview of trends without overwhelming the dataset, while event-based recordings ensured the capture of all relevant fluctuations. Data collected during nursing care or procedures—as indicated in the ‘report form’—were excluded from the analyses to minimize confounding.

Given the high possibility of motion artifacts and electrode disconnections in neonatal intensive care settings, the identification of tachycardia, tachypnea, and desaturation events did not rely solely on threshold values or duration. Instead, all episodes were carefully verified by two experienced neonatal intensive care researchers through visual inspection of the ECG and respiratory waveforms, as well as clinical context, to distinguish true physiological events from artifacts. True events were characterized by continuous, artifact-free signals, allowing for explicit recognition of QRS complexes for tachycardia, consistent respiratory patterns for tachypnea, and stable oxygen saturation waveforms for desaturation.

#### 2.2.3. Confounding and Correlated Variables

Several types of data were recorded during the study procedures to allow data analysis and control for confounding variables.

For each enrolled preterm newborn, a data form was completed, including varied demographic information (e.g., gestational age at birth and enrollment, weight at birth and enrollment, and sex).

During observations, the nurses assigned to the enrolled preterm newborns were required to complete a ‘report form’ to indicate when nursing care or other procedures (e.g., blood sampling, kangaroo mother care) were performed. In addition, the research group also recorded any respiratory support, the location of the hospital stay (in terms of room and incubator or bed), the administration of medication, and the time of recording (morning or evening).

### 2.3. Data Analysis

Firstly, we performed a descriptive analysis to assess the trend of the collected data. We analyzed the mean environmental noise levels, changes in vital parameters, and demographic characteristics of the enrolled neonates.

We then explored our data using a scatterplot graph to analyze the relationship between the variables. As a result, we performed a Kendall τ-b correlation analysis between the variables considered relevant to our objective (dB with heart rate, dB with respiratory rate, and dB with oxygen saturation).

Finally, since we found a statistically significant correlation between dB and heart rate, we dichotomized the variable of tachycardia (“YES” with a heart rate ≥ 180 bpm or “NO” with a heart rate < 180 bpm) and started a logistic regression model with all the variables included in order to better understand the correlation identified.

### 2.4. Ethical Consideration

The Regional Ethics Committee of Liguria approved the study on 14 March 2022 (protocol number 417/2021–DB ID 11651).

A member of the research team provided a detailed explanation of the study to the caregivers of all preterm newborns meeting the inclusion criteria, and written informed consent was obtained prior to participation.

## 3. Results

### 3.1. Description of Environmental Noises

**Figure 1:**
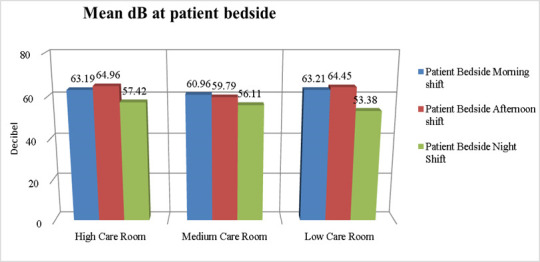
dB levels at the patient’s bedside in different NICU rooms.

**Figure 2:**
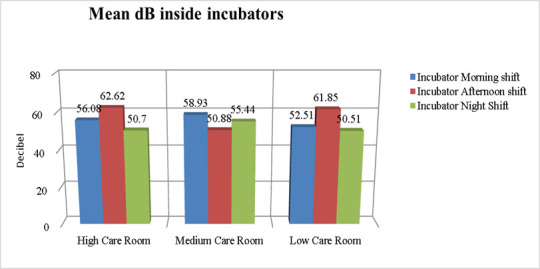
dB levels inside the incubators in different NICU rooms.

Figure 1 and Figure 2 show the mean dB of environmental noise recorded in the different NICU rooms at various times of day, both at the patient’s bedside and inside the incubators. The recorded environmental noise was always greater than 45 dB. The Student’s *t*-test shows no statistically significant difference in the mean of recorded environmental noise.

### 3.2. Patients’ Characteristics

We enrolled six preterm newborns, representing 35.3% of the eligible patients during the study period. Each patient was observed for a minimum of one to a maximum of seven registrations, for a total of twenty-two registrations over 1770 min of observation. Each registration lasted from a minimum of 1 h and 30 min to a maximum of 4 h.

Table 1 presents the characteristics of the preterm newborns enrolled in the study, as well as the characteristics of the registrations.

Table 2 reports a summary of vital parameter alterations registered during recording.

### 3.3. Correlation Analysis Results

In our database, we recorded 520 punctual events, which refer to a specific minute. Each event includes measurement of dB, heart rate, respiratory rate, and oxygen saturation, all recorded during our 22 registrations.

The data exploration reveals a non-linear relationship between the variables analyzed (dB, heart rate, respiratory rate, and oxygen saturation). Then, as we obtained a small dataset, we decided to perform a Kendall correlation to evaluate the existence of a relationship between our variables [17] (Sheskin, 2004) (see results in Table 3). The Kendall τ-b between dB and heart rate (τ-b = 0.089, *p* = 0.003, n = 520) suggests a monotonic crescent tendency between these two variables, even if the relationship is not strong, although it is statistically significant. The other relationships studied are not statistically significant, except for the correlation between respiratory and heart rates (τ-b = −0.244, *p* < 0.001, n = 520).

The same data analysis clustered for every patient shows statistically significant results for some patients and no significance for others, suggesting an elevated individual fluctuation (Supplementary Materials S1).

### 3.4. Logistic Regression Analysis Results

We found a weak relation between heart rate and dB, which we explored using logistic regression analysis. We dichotomized the heart rate data as follows: a heart rate > 180 bpm = YES (the patient experienced a tachycardia event) and a heart rate < 180 bpm = NO (the patient did not experience a tachycardia event).

The first model we analyzed included only the sound level measured in decibels (dB) as a predictor of the probability of tachycardia without including any additional explanatory variables. This approach was intended to serve as a baseline model, allowing us to evaluate the predictive power of noise exposure alone. The model’s performance was assessed using the Akaike Information Criterion (AIC), which yielded a value of 686, indicating a relatively poor fit. Additionally, the model’s coefficient of determination (R^2^) was 0.0274, meaning that the dB level accounted for only 2.7% of the variability observed in tachycardia occurrences. These results suggest that noise level alone has limited explanatory power for predicting tachycardia, highlighting the need to consider other relevant variables in subsequent models [18].

The final model that best explains our data yields an AIC of 385, suggesting that our model is not overly complicated, and a McFadden R-squared of 0.242, which explains 24.2% of the variability in tachycardia, indicating a good fit of this model to our data [19]. In this model, we include all covariates that could potentially impact heart rate in our population: gestational age at birth and enrollment, weight at birth and enrollment, presence or absence of respiratory support, sex, patient’s setting (incubator or bed), respiratory rate, and oxygen saturation.

The results of this model are presented in Table 4.

The logistic regression model shows that dB has a significant effect (*p* < 0.001): each 1 dB increase in noise is associated with a 6.6% increase in the odds of tachycardia.

Respiratory rate (*p* < 0.001) also significantly affects this model. The odds ratio of 0.959 suggests that the probability of tachycardia decreases by approximately 4.1% for each unit increase in respiratory rate.

The other variables analyzed did not significantly affect the probability of tachycardia events.

We further investigated whether the effect of sound level (dB) on tachycardia risk might differ depending on respiratory rate by including an interaction term between these variables in the model (see Table 5). The interaction term tests whether the combined effect of dB and respiratory rate differs from the sum of their individual additive effects. However, this interaction was not statistically significant.

## 4. Discussion

Our results suggest that increased environmental noise is associated with a higher risk of tachycardia events in preterm newborns admitted to NICUs. It is important to note that the enrolled infants had a relatively high gestational age (mean 35 + 2 weeks) as a result of the study’s inclusion and exclusion criteria, which excluded more premature and clinically unstable cases. Although the correlation is not strong, it is statistically significant and further confirmed by the logistic regression model, which indicates that for every 1 dB increase in environmental noise, the risk of tachycardia increases by 6.6%. This increase means that a 10 dB increase is associated with a 66% rise in risk. While this figure may seem modest in absolute terms, it is highly clinically relevant for the vulnerable population of preterm newborns.

These findings are consistent with the existing literature, which has documented the impact of environmental noise on newborns’ vital parameters [14,15]. Notably, our study stands out for its innovative methodological approach: the synchronized video recording of vital parameters, combined with punctual environmental noise measurements, enables a high-resolution dataset that enhances the accuracy and granularity of analysis. This technique reduces the risk of misclassification and enhances the reliability of the identified associations [20].

Our findings underscore the urgent need to implement effective noise reduction strategies in NICUs. While architectural changes—such as the introduction of single-family rooms—have proven effective in reducing noise levels [8], these solutions may not be feasible in many settings due to structural or financial constraints. In such cases, other evidence-based interventions can be both feasible and impactful. Thirumalesh and Thekkanath [2] demonstrated the effectiveness of using earplugs to mitigate noise exposure among preterm infants. Studies by Wang and colleagues [5] and Ahamed and colleagues [4] highlighted the benefits of staff education, behavioral adjustments, and noise awareness campaigns. Additionally, some studies have explored using white noise to buffer procedural sounds in NICUs, showing promising effects on stabilizing vital parameters [21].

These interventions, many of which are low-cost and non-invasive, hold significant potential in minimizing the harmful physiological effects of noise exposure—such as the increased risk of tachycardia observed in our study. The consistent application of such strategies can help create a quieter, more developmentally supportive environment in the NICU, benefiting both infants and healthcare providers [22].

An interesting finding in our analysis is the inverse relationship between respiratory rate and the risk of tachycardia. It is plausible to hypothesize the existence of a compensatory physiological mechanism whereby an increase in respiratory rate improves tissue oxygenation, thereby helping to regulate heart rate. Another possible explanation could relate to increased tidal volume, which may activate parasympathetic pathways and reduce heart rate. In adult populations, complex interactions between respiratory rate, heart rate, and the autonomic nervous system have been described [23]. Pediatric studies, such as those by Amichai and colleagues [24], have also reported that reduced respiratory rates may lead to elevated heart rates due to heightened sympathetic tone in specific patient groups.

Although the interaction term between decibel levels and respiratory rate was not statistically significant (*p* = 0.158), its negative coefficient suggests a potential moderating effect, wherein higher respiratory rates buffer the adverse impact of noise on heart rate regulation. This regulation is particularly relevant in preterm newborns whose autonomic nervous systems are not yet fully developed [25]. This observation aligns with existing physiological models, which suggest that increased ventilation may mitigate autonomic arousal in response to stressors [26]. However, due to the lack of statistical significance, these findings should be interpreted cautiously and warrant further investigation in larger or stratified samples.

### Study Limitations

Despite the strengths of our methodological approach, this study presents several limitations. First, the small sample size (n = 6), while partially offset by the high number of recorded events (n = 520), limits the generalizability of the findings. Nevertheless, the richness of the event-based dataset offers a solid foundation for exploratory analysis.

Secondly, although we attempted to control for confounding variables, some potentially influential factors—such as behavioral state, sleep quality, and pain—were not assessed.

A further limitation concerns the statistical modeling. Repeated measurements per patient were treated as independent data points, which does not fully account for intra-subject correlation. Moreover, the final logistic regression model, although statistically significant, included a relatively high number of covariates, given the small sample size, which raises the risk of overfitting and limits the robustness of coefficient estimates.

Vital parameters were obtained from monitor displays, which reflect calculated values rather than raw physiological signals. These are typically derived through algorithmic averaging over short time windows, which may introduce a delay in the representations of responses. This technical constraint limits the precision of time-dependent associations. Furthermore, episodes of tachycardia triggered by noise may activate acoustic alarms, which in turn increase environmental noise, potentially exacerbating infant stress in a feedback loop.

Finally, while the model explained approximately 24% of the variability in tachycardia events, a substantial portion remains unexplained. This result suggests the likely influence of additional, unmeasured physiological or environmental factors. As this was a pilot observational feasibility study, causal relationships between noise exposure and alterations in vital parameters cannot be established.

Future studies should include larger and more diverse newborn populations and adopt longitudinal or hierarchical modeling strategies to improve the validity and reliability of results. These efforts will enable a more comprehensive understanding of how environmental noise impacts newborns’ physiological regulation in the NICU setting.

## 5. Conclusions

Our findings suggest that environmental noise, measured in decibels, is a significant variable influencing the likelihood of tachycardia in neonates admitted to NICUs. Even modest increases in decibel levels were associated with a meaningful rise in the risk of tachycardic episodes, highlighting the clinical importance of environmental monitoring and noise management in neonatal care. The respiratory rate also emerged as a significant variable in the multivariate model, indicating a complex interplay between different physiological responses to stressors in this vulnerable population.

Although our final regression model accounted for approximately 24% of the variability in tachycardia events, this suggests that while environmental noise is a key contributing factor, it is not the sole determinant of tachycardia events. Other physiological and contextual variables—such as gestational age, birth weight, and clinical status—likely modulate an infant’s susceptibility to noise-induced stress.

Our findings support the incorporation of noise reduction strategies and individualized monitoring protocols in NICU settings. The next RCT phase will allow further evaluation of targeted interventions.

## Figures and Tables

**Table 1:** Patients and registration characteristics.

Patients’ Characteristics (N = 6)	% (N)	Mean (SD)	Min	Max
Gender Female	83 (5)	-	-	-
*Gestational age at birth (weeks + days)*	-	29 + 4 (±4.19 + ±1.25)	24 + 4	37 + 3
*Gestational age at enrollment (weeks + days)*	-	35 + 4 (±1.28 + ±1.96)	33 + 1	38 + 2
*Weight at birth (grams)*	-	1078 (±0.375)	700	2010
*Weight at enrolment (grams)*	-	1651 (±0.084)	1580	1960
***Registrations’ characteristics (N = 22)***				
**Respiratory support**		-	-	-
None	18 (4)			
High Flow Nasal Cannula (HFNC)	82 (18)			
**Patient accommodation**		-	-	-
Incubator	36 (8)			
Bed	64 (14)			
**Time of registration**		-	-	-
Morning	73 (16)			
Afternoon	27 (6)			
**Decibel**	-	59.61 (±4.56)	44.2	98

**Table 2:** Summary of vital parameters alteration.

Event Type	Patients Affected	Definition	Mean Events Per Patient (Range)
*Tachycardia*	5	Heart rate > 180 bpm	1.5–28 events
*Tachypnoea*	3	Respiratory frequency > 60 acts/min	7–90 events
*Desaturation*	5	SpO_2_ < 90%	1–72.5 events

**Table 3:** Kendall τ-b results.

		dB	Heart Rate	Respiratory Rate	Oxygen Saturation
**dB**	Kendall Tau B *p*-value	— __			
**Heart Rate**	Kendall Tau B *p*-value	0.089 0.003	—		
**Respiratory Rate**	Kendall Tau B *p*-value	−0.048 0.184	−0.244 <0.001	— __	
**Oxygen Saturation**	Kendall Tau B *p*-value	−0.021 0.512	0.024 0.445	−0.031 0.414	—— __

**Table 4:** Results of logistic regression model—tachycardia YES/NO.

Predictor	Estimate	SE	Z	*p*	Odds Ratio
*Intercept*	528.51041	23,494.94410	0.0225	982	3.38 × 10^229^
*dB*	0.06408	0.01629	3.9335	<0.001	1.066
*Gestational Age_birth*	−7.00293	336.38270	−0.0208	983	9.09
*Gestational Age_enrollment*	−7.88762	324.57478	−0.0243	981	3.75
*Weight_birth*	0.12254	5.73118	0.0214	983	1.130
*Weight_enrollment*	−0.11167	5.18398	−0.0215	983	0.894
*Gender*	−89.03205	4144.16798	−0.0215	983	2.16 × 10^−39^
*Ventilation*	0.44514	0.42228	1.0541	292	1.561
*Patient unit*	−0.52062	0.59322	−0.8776	380	0.594
*Respiratory rate*	−0.04215	0.00777	−5.4247	<0.001	0.959
*Saturation*	0.00569	0.01741	0.3271	744	1.006

**Table 5:** Results of a logistic regression model with an interaction term.

Predictor	Estimate	SE	Z	*p*	Odds Ratio
*Decibel*	0.13657	0.0504	2.710	0.007	1.14634
*Respiratory Rate*	0.03786	0.0611	0.620	0.535	1.03858
*DecibelsXRespiratoryRate*	−0.00136	9.64 × 10^−4^	−1.413	0.158	0.99864

## Data Availability

The raw data supporting the conclusions of this article will be made available by the authors upon request.

## Supplementary Materials

The following supporting information can be downloaded at: https://www.mdpi.com/article/10.3390/children12060757/s1, Table S1: Patient ID DN1; Table S2: Patient ID GN1; Table S3: Patient ID LF1; Table S4: Patient ID CR1; Table S5: Patient ID DA1.

## Abbreviations

The following abbreviations are used in this manuscript:

MDPI	Multidisciplinary Digital Publishing Institute
DOAJ	Directory of open access journals
TLA	Three-letter acronym
LD	Linear dichroism
NICU	Newborn Intensive Care Unit
dB	Decibel
