# Supplementary material for: Not a Quiet Place: Understanding Noise Level in a Newborn Intensive Care Unit (NICU) and Its Relation with Newborn’s Vital Parameters, a Pilot Feasibility Study
Source: Children (Basel). 2025 Jun 11;12(6):757. doi: 10.3390/children12060757 (PMC12191224; doi:10.3390/children12060757)
Supplement: Supplementary file 1 [file children-12-00757-s001.zip › children-3647002-supplementary.pdf]

Supplementary Material n. 1 - Kendall τ-b calculated on single patient

N. 1 – Patient ID DN1

		Decibels	Heart Rate	Respiratory Rate	Oxygen Saturation
**Decibels**	Kendall Tau B	—			
	p value	—			
**Heart Rate**	Kendall Tau B	-0.117	—		
	p value	0.037			
**Respiratory Rate**	Kendall Tau B	-0.034	0.097	—	
	p value	0.525	0.091	—	
**Oxygen Saturation**	Kendall Tau B	0.233	-0.257	-0.138	——
	p value	< .001	< .001	0.011	—

N. 2 – Patient ID GN1

		Decibels	Heart Rate	Respiratory Rate	Oxygen Saturation
**Decibels**	Kendall Tau B	—			
	p value	—			
**Heart Rate**	Kendall Tau B	0.120	—		
	p value	0.033			
**Respiratory Rate**	Kendall Tau B	-0.157	-0.013	—	
	p value	0.085	0.888	—	
**Oxygen Saturation**	Kendall Tau B	0.143	0.013	0.023	——
	p value	0.023	0.840	0.820	—

N. 3 – Patient ID LF1

		Decibels	Heart Rate	Respiratory Rate	Oxygen Saturation
**Decibels**	Kendall Tau B	—			
	p value	__			
**Heart Rate**	Kendall Tau B	-0.229	—		
	p value	0.026			
**Respiratory Rate**	Kendall Tau B	0.363	-0.149	—	
	p value	< .001	0.149	__	
**Oxygen Saturation**	Kendall Tau B	0.431	-0.296	0.418	——
	p value	< .001	0.006	< .001	__

N. 4 – Patient ID CR1

		Decibels	Heart Rate	Respiratory Rate	Oxygen Saturation
**Decibels**	Kendall Tau B	—			
	p value	__			
**Heart Rate**	Kendall Tau B	0.033	—		
	p value	0.698			
**Respiratory Rate**	Kendall Tau B	0.116	-0.119	—	
	p value	0.177	0.172	__	
**Oxygen Saturation**	Kendall Tau B	-0.253	0.176	-0.389	——
	p value	0.007	0.060	< .001	__

N. 5 – Patient ID DA1

		Decibels	Heart Rate	Respiratory Rate	Oxygen Saturation
**Decibels**	Kendall Tau B	—			
	p value	—			
**Heart Rate**	Kendall Tau B	0.149	—		
	p value	0.126			
**Respiratory Rate**	Kendall Tau B	0.107	0.047	—	
	p value	0.630	0.835	—	
**Oxygen Saturation**	Kendall Tau B	-0.085	-0.188	0.145	——
	p value	0.435	0.088	0.550	—

N. 6 – Patient ID AV1

		Decibels	Heart Rate	Respiratory Rate	Oxygen Saturation
**Decibels**	Kendall Tau B	—			
	p value	—			
**Heart Rate**	Kendall Tau B	-0.059	—		
	p value	0.765			
**Respiratory Rate**	Kendall Tau B	—	—	—	
	p value	—	—	—	
**Oxygen Saturation**	Kendall Tau B	-0.351	-0.370	—	——
	p value	0.098	0.086	—	—
